# Assisted Living Administrators' Views of Palliative Care

**DOI:** 10.1093/geroni/igab046.3259

**Published:** 2021-12-17

**Authors:** Elise Abken, Molly Perkins, Alexis Bender

**Affiliations:** 1 Emory University School of Medicine, Atlanta, Georgia, United States; 2 Emory University of Medicine, Atlanta, Georgia, United States; 3 Emory University, Atlanta, Georgia, United States

## Abstract

As many older adults with progressive chronic conditions choose to age-in-place in assisted living (AL) communities, external healthcare workers (e.g., those who provide palliative care) increasingly support AL staff in caring for residents with complex health needs. Palliative care is a branch of healthcare dedicated to preserving quality of life by attending to the physical, mental, and spiritual needs of individuals with chronic, life-threatening diseases and is well suited to manage AL residents’ progressive medical conditions. However, AL residents and their care partners often face barriers to accessing palliative care. Using data from a larger 5-year NIA-funded study, we examined AL administrator knowledge and use of palliative care in seven AL communities around the Atlanta metropolitan area that were racially, ethnically, and socioeconomically diverse. Findings from thematic analysis of semi-structured interviews with 16 administrators indicated that 15 of 16 administrators were familiar with palliative care. A minority of administrators clearly distinguished palliative care from hospice services and conceptualized it as a “bridge” to hospice services. Administrators emphasized how palliative care assists communities in caring for health concerns in-house rather than having to send residents to the hospital. Despite their positive view of palliative care, administrators described infrequent use of palliative services in their communities. Findings show that although none of the AL communities integrate palliative care with their service offerings, AL administrators see value in palliative care for their residents. We provide recommendations for improving palliative care access and quality of life for AL residents at end of life.

